# Venous Leg Compression for Tissue Decongestion in Patients With Worsening Congestive Heart Failure

**DOI:** 10.3389/fcvm.2022.847450

**Published:** 2022-07-08

**Authors:** Jose Civera, Gema Miñana, Rafael de la Espriella, Enrique Santas, Clara Sastre, Anna Mollar, Adriana Conesa, Ana Martínez, Eduardo Núñez, Antoni Bayés-Genís, Julio Núñez

**Affiliations:** ^1^Cardiology Department, Hospital Clínico Universitario de Valencia, INCLIVA Instituto de Investigación Sanitaria, Valencia, Spain; ^2^Department of Medicine, Universitat de València, Valencia, Spain; ^3^CIBER in Cardiovascular Diseases (CIBERCV), Madrid, Spain; ^4^Heart Failure Unit, Cardiology Department, Hospital Universitari Germans Trias i Pujol, Badalona, Spain; ^5^Department of Medicine, Autonomous University of Barcelona, Barcelona, Spain

**Keywords:** worsening heart failure, congestion, diuretic efficiency, inferior vena cava, venous leg compression

## Abstract

**Aims:**

Venous leg compression (VLC) with elastic bandages has been proposed as a potentially useful strategy for decreasing tissue congestion. We aimed to evaluate the effect of VLC on short-term changes on intravascular refill, assessed by inferior vena cava (IVC) diameter in patients with worsening heart failure (WHF) requiring parenteral furosemide. Additionally, we sought to evaluate whether early changes in IVC were related to short-term decongestion.

**Methods:**

This is a prospective study in which we included 20 consecutive ambulatory patients with WHF treated with subcutaneous furosemide and VLC for at least 72 h. The endpoints were (a) short-term changes in IVC, (b) the association between decongestion and 3-h IVC changes following VLC. Changes in continuous endpoints and their longitudinal trajectories were estimated with linear mixed regression models. All analyses were adjusted for multiple comparisons.

**Results:**

Following administration of subcutaneous furosemide and VLC, we found a significant increase in 3-h IVC diameter (ΔIVC = 1.6 mm, CI 95%: 0.7–2.5; *p* < 0.001), with a greater increase in those with baseline IVC≤21 mm (2.4 vs. 0.8 mm; *p* < 0.001). 3-h intravascular refill (increase in IVC≥2 mm) was associated with greater decongestion (natriuresis, weight, peripheral edemas, and dyspnea) in those with baseline IVC≤21 mm but not when IVC>21 mm (*p* < 0.05 for all comparisons).

**Conclusions:**

In this cohort of patients with congestive WHF treated with subcutaneous furosemide and VLC, we found a greater increase in short-term IVC in those with IVC ≤21 mm at baseline. In this subset of patients, a 3-h increase in IVC≥2 mm was associated with greater short-term decongestion.

## Introduction

Fluid overload explains most of the symptoms and signs of patients with worsening heart failure (WHF) (1, 2). Diuretics constitute the mainstay armamentarium in these patients, although the evidence endorsing the optimal diuretic strategy (intensity and sequence of the diuretic prescription) is scarce (3, 4). Optimal decongestion should imply tissue and vascular decongestion. However, at least in the short-term, most used interventions, such as parenteral diuretics, have a predominant effect by reducing intravascular congestion (3, 4). Several strategies have been postulated to mobilize extravascular volumes, such as infusion of loop diuretics and hypertonic solutions (sodium or albumin), without consistent evidence about their utility (5, 6). Venous leg compression (VLC) by using elastic bandages has been proposed as another potentially useful strategy for decreasing tissue congestion. However, its efficacy and safety in heart failure (HF) patients require a profound evaluation (7).

In this work, we sought to evaluate the association between VLC and short-term changes in intravascular refill and whether these changes are related to decongestion parameters in patients with WHF that require parenteral furosemide administration.

## Materials and Methods

### Study Design and Eligibility Criteria

This is a one-arm open-label prospective study in which we included 20 consecutive ambulatory patients with WHF treated with subcutaneous furosemide and VLC for at least 72 h between January 1st, 2020, and June 1st, 2021, at an outpatient HF-Clinic in Spain (Hospital Clinic Universitari, Valencia-Spain). All patients received a subcutaneous furosemide infusion for the treatment of WHF. Patients were eligible if they presented with WHF with peripheral edema (at least grade 1+) that required parenteral ambulatory administration of furosemide. All patients had an established diagnosis of HF according to ESC guidelines (8). Exclusion criteria consisted of (a) acute decompensated HF requiring hospital admission (acute pulmonary edema, evidence of hypoxemia, defined as an oxygen saturation <90% in pulse oximetry or oxygen partial pressure <80 mmHg in arterial blood gas analysis), (b) cardiogenic shock, (c) symptomatic hypotension or any systolic blood pressure (SBP) <90 mmHg, and d) index event triggered by an uncontrolled arrhythmia (advanced heart block without a pacemaker, sustained ventricular tachycardia, therapeutic defibrillator shock, or atrial fibrillation/flutter with sustained ventricular response >150 bpm), infection/sepsis, or severe anemia (hemoglobin <7 g/dL), and patient that require hospitalization at clinician judgment. Patients on renal replacement therapy or ultrafiltration were also excluded. This study complied with the Declaration of Helsinki and was approved by the local institutional review committees. All patients signed an informed consent form.

### Procedures

#### Subcutaneous Administration of Furosemide

Subcutaneous furosemide was administered by using a single-use, continuous infusion pump system (DOSI-FUSER^®^, Leventon, S.A.U, Barcelona, Spain) and a standard commercial subcutaneous infusion set. The infusion pump system consists of an elastomeric balloon inside a rigid container, an infusion line with the capillary device, and a Luer-lock connector that attaches to the standard subcutaneous infusion set. After the balloon is inflated, the medication flows through the capillary device due to the pressure from the elastomeric balloon, which determines the flow rate. We used an infusion pump containing a 250 mL balloon reservoir with a nominal continuous flow rate of 2.1 mL/h over 72 h.

Subcutaneous furosemide dose was calculated based on the subject's outpatient oral dose using a 1:1.25 conversion (80 mg of oral furosemide = 100 mg of subcutaneous furosemide). Therefore, for administering a daily dose of 100 mg of subcutaneous furosemide, a 2 mg/mL drug concentration was required (dilution: 500 mg of non-formulated furosemide in 250 mL of 0.9% sodium chloride). Specialized HF nurses filled the infusion system following the manufacturer's instructions, placed the subcutaneous catheter, and thoroughly explained general guidelines and troubleshooting to study participants.

#### Venous Leg Compression

Compression of the lower limbs was performed with a multi-component layer compression bandage system at a pressure of 20 mmHg (UrgoK2 LITE^®^) consisting of two dynamic components: an inelastic and elastic bandage. When combined, the two layers constitute one compression bandaging system that provides both a dynamic static stiffness profile and tolerable resting pressures. The first layer, KTech^®^, is an inelastic bandage (approximately 75% extensibility), consisting of viscose and polyester wadding with a knitted layer made of polyamide and elastane. When in contact with the skin, the KTech^®^ layer distributes the pressure uniformly over the surface of the leg and provides compression, along with protection and absorbency when needed (9). Ktech^®^ provides a high working pressure with a low resting pressure, which in combination with the action of the calf muscle creates a massage effect, assisting venous return and reducing edema levels. The second layer, KPress^®^, is an elastic cohesive bandage of approximately 160% extensibility, made from synthetic components, such as acrylic, polyamide, and elastane. This outer bandage provides the additional compression necessary to achieve the required therapeutic pressure and, more critically, maintains the recommended resting pressures necessary to maintain improved blood flow (9). These pressures are consistently maintained over time (during 7 days) (10). Each bandage layer displays an oval indicator (the PresSure^®^ system, also known as the etalonnage) that expands into a circle when stretched correctly. Proper application is further enhanced by guides for appropriate overlap of layering. There are two different sizes of UrgoK2 LITE^®^ according to the ankle perimeter (18–25 or 25–32 cm). For each patient, the ankle perimeter was measured, and the right size was selected.

#### Assessment of Inferior Vena Cava Diameter

The inferior vena cava (IVC) diameter was visualized by echography (11), with patients in the supine position, using subcostal 4 chamber view (midline, inferior to the xiphoid, angling to the right). The maximum IVC diameter during the respiratory cycle was measured approximately 3 cm before the merger with the right atrium. An IVC maximum diameter of >21 mm was defined as dilated IVC. IVC diameter was evaluated at baseline and 3, 24, 48, and 72 h after applying the compression bandage. A change in IVC at 3-h >2 mm was considered significant.

#### Clinical Monitoring and Biomarkers Assessment

All patients were physically visited on the day of presentation, at 24, 48, and 72-h. At these encounters, we registered the New York Heart Association (NYHA) class, pedal edema, weight, and vital signs. The pedal edema was assessed by 1+ to 4+ grade (grade 1+: slight pitting 2 mm depth, grade 2+: somewhat deeper pit 4 mm, grade 3+: noticeably deep pit 6 mm, and grade 4+: very deep pit 8 mm), and by measuring the diameter of lower limbs 10 cm above the external malleolus. The mean diameter between both limbs was registered.

We also assessed dyspnea visual analog scale (VAS) and standard plasma laboratory data [estimated glomerular filtration rate (eGFR), plasma electrolytes (sodium and potassium), and amino-terminal pro-brain natriuretic peptide (NT-proBNP)] at presentation and 72-h. The dyspnea VAS scale of 0 corresponds to the patient's subjective feeling of “I can breathe normally,” and a dyspnea VAS score of 10 corresponds to “I can't breathe at all.” Spot urinary sodium was assessed each 24-h after treatment intervention, at 24, 48, and 72 h.

### Endpoints

The endpoints were: a) changes in short-term IVC following administration of subcutaneous furosemide and VLC, and b) the relationship between 3-h changes in IVC and parameters of decongestion (natriuresis, pedal edema, weight, dyspnea VAS, and NT-proBNP). In addition, safety endpoints included changes in eGFR, SBP, electrolytes, and the proportion of patients that symptomatically did not tolerate the 72-h leg venous compression.

### Statistical Analysis

Continuous baseline variables were expressed as median [interquartile interval (IQI)]. Discrete variables were presented as numbers (percentages). Changes in continuous endpoints and their longitudinal trajectories were estimated with linear mixed regression models (LMRMs). Continuous exposures with a non-parametric distribution were log-transformed [NT-proBNP (lnNT-proBNP)]. Multivariate estimates were adjusted for age, sex, baseline eGFR, left ventricular ejection fraction (LVEF), and the baseline endpoint value regardless of their *p*-value. The LMRMs are presented as least square means (LSM) with their respective 95% confidence intervals. *P*-values were adjusted for multiple comparisons (Sidak procedure). A 2-sided *p*-value of < 0.05 was set as a criterion for statistical significance. All analyses were performed in Stata 15.1 (Stata Statistical Software, Release 15 [2017]; StataCorp LP, College Station, TX, USA).

## Results

The median age was 80 years (73–85), 8 (40%) patients were female, 15 (75%) showed a prior history of NYHA III, and all of them were previously admitted for acute HF, all of them were on treatment with oral loop diuretics [median furosemide equivalent doses 80 mg/day (40–120)] and showed peripheral edema at presentation (90% with grades 3+ to 4+). The median (p25–p75%) SBP, heart rate, eGFR, NT-proBNP and carbohydrate antigen (CA125) at presentation were 127 mmHg (110–139), 74 bpm (64–82), 44 ml/min/1.73 m^2^ (33–61), 2,738 pg/ml (1,290–8,585), and 34 U/mL (15–125), respectively. The median (p25–p75%) of LVEF and tricuspid annular plane systolic excursion (TAPSE) were 52% (36–60) and 17.5 mm (14–19), respectively. A total of 12 (60%) patients had LVEF ≥50%. The median (p25–p75%) of IVC diameter was 22.5 mm (15–27). Half of the patients displayed IVC diameter ≤21 mm. Patients were treated with homogenous doses of subcutaneous furosemide [median 100 mg/day (min: 80, and max: 120)].

Baseline characteristics across IVC status (≤21 vs. >21 mm) are summarized in Table 1. Patients with IVC ≤21 mm showed lower NYHA class, NT-proBNP, and jugular engorgement (Table 1). There were no differences in the severity of peripheral edema or other clinical parameters of congestion.

**Table 1:** Baseline characteristics.

	**Total population (*N* = 20)**	**IVC at baseline ≤21 mm (*N* = 10)**	**IVC at baseline >21 mm (*N* = 10)**	***p*-value**
**Demographics and medical history**				
Age, years	80.0 (72.5–84.5)	82.0 (68.0–85.0)	78.0 (74.0–83.0)	0.705
Male, *n* (%)	12 (60.0)	5 (50.0)	7 (70.0)	0.650
Hypertension, *n* (%)	17 (85.0)	9 (90.0)	8 (80.0)	1.000
NYHA class, *n* (%) II	5 (25.0)	5 (50.0)	0
III	15 (75.0)	5 (50.0)	10 (100.0)
Diabetes mellitus, *n* (%)	13 (65.0)	7 (70.0)	6 (60.0)	1.000
Weight, Kg	83.0 (77.4–89.5)	80.2 (77.3–89.0)	85.4 (77.5–89.9)	0.597
COPD, *n* (%)	3 (15.0)	1 (10.0)	2 (20.0)	1.000
Renal failure, *n* (%)	14 (70.0)	8 (80.0)	6 (60.0)	0.628
Atrial fibrillation, *n* (%)	15 (75.0)	6 (60.0)	9 (90.0)	0.303
**Vital signs and physical examination**				
SBP, mmHg	127 (110–138)	123 (102–137)	129 (112–140)	0.406
DBP, mmHg	70.5 (62.5 −75.5)	69.5 (65–73)	71 (60–78)	0.597
Heart rate, bpm	73.5 (64–2)	68.5 (62–75)	81 (70–86)	0.059
Peripheral edema, *n* (%)			
1+ (slight)	2 (10.0)	1 (10.0)	1 (10.0)
2+ (moderate)	0	0	0
3+ (marked) 12 (60.0)	6 (60.0)	6 (60.0)
4+ (serious)	3 (15.0)	1 (10.0)	3 (30.0)
Pleural effusion, *n* (%)	3 (15.0)	1 (10.0)	2 (20.0)	1.00
Jugular engorgement, *n* (%)	15 (75.0)	5 (50.0)	10 (100.0)	0.033
Lower limb perimeter, cm	27.5 (25.5 – 29.5)	26.8 (25.0 – 29.0)	28.0 (26.0 – 29.5)	0.438
**Echocardiography**				
LVEF, %	51.5 (36–60)	55 (37–60)	48 (35–60)	0.678
PASP, mmHg	44.5 (35–50)	39.5 (35–45)	50 (35–52)	0.109
TAPSE, mm	17.5 (14–19)	17 (15–19)	18 (14–21)	1.000
Inferior vena cava, mm	22.5 (14.5–27)	14.5 (14–20)	27 (25–28)	<0.001
**Laboratory tests**				
Serum sodium, mmol/L	139.5 (137–142.5)	140.5 (139–143)	138 (137–142)	0.212
Serum potassium, mmol/L	4.4 (4.1–4.6)	4.5 (4.3–4.7)	4.3 (4.0–4.5)	0.102
eGFR, mL/min/1.73 m2	31.3 (23.0–40.7)	23.0 (15.1–48.8)	37.3 (25.3–40.7)	0.513
Hematocrit, %	35 (31–43)	37 (31–43)	35 (33–41)	0.775
Urine creatinine mmol/L	68 (43–94)	73 (65–94)	39.5 (27–95.5)	0.131
Urine sodium, mmol/L	64 (49–86)	64 (56–83)	64 (33–87)	0.935
Urine potassium mmol/L	35.5 (28–45)	35 (31–42)	37 (23–51)	0.894
NT-proBNP, pg/mL	2738 (1290–8585)	1950 (880–4246)	8585 (2588–11765)	0.018
CA125, U/mL	33.5 (15–125)	32 (15–379)	34 (14.5–87)	0.790
**Pharmacological heart failure therapy at baseline**				
Loop diuretics (oral), *n* (%)	20 (100)	10 (100)	10 (100)	1.000
FED, mg	80 (40–80)	70 (40–80)	80 (80–120)	0.186
Furosemide sc dose, mg	100 (90–120)	100 (80–100)	110 (100–120)	0.054
Chlorthalidone, *n* (%)	13 (65.0)	4 (40.0)	9 (90.0)	0.057
Acetazolamide, *n* (%)	1 (5.0%)	0	1 (10.0)	1.000
MRA, n (%)	12 (60.0)	4 (40.0)	8 (80.0)	0.170
Sacubitril-valsartan, *n* (%)	6 (30.0)	3 (30.0)	3 (30.0)	1.000
ACEI/ARB, *n* (%)	7 (35.0)	4 (40.0)	3 (30.0)	1.000
iSGLT-2, *n* (%)	9 (45.0)	5 (50.0)	4 (40.0)	0.656
Betablockers, *n* (%)	18 (90.0)	9 (90.0)	9 (90.0)	1.000

*ACEI, angiotensin converting enzyme-inhibitors; ARB, angiotensin receptor blockers; CA125, carbohydrate antigen 125; COPD, chronic obstructive coronary disease; DBP, diastolic blood pressure; eGFR, estimated glomerular filtration rate; FED, furosemide equivalent dose; iSGLT-2, sodium-glucose co-transporter-2 inhibitors; LVEF, left ventricle ejection fraction; MRA, mineralcorticoid receptor antagonists; NT-proBNP, amino-terminal pro-brain natriuretic peptide; NYHA, New York Heart Association; PASP,: pulmonary arterial systolic pressure; SBP, systolic blood pressure; TAPSE, tricuspid annular plane systolic excursion*.

*Continuous variables are expressed as median (p25–p75%)*.

### Changes in IVC Following Venous Leg Compression

**Figure 1:**
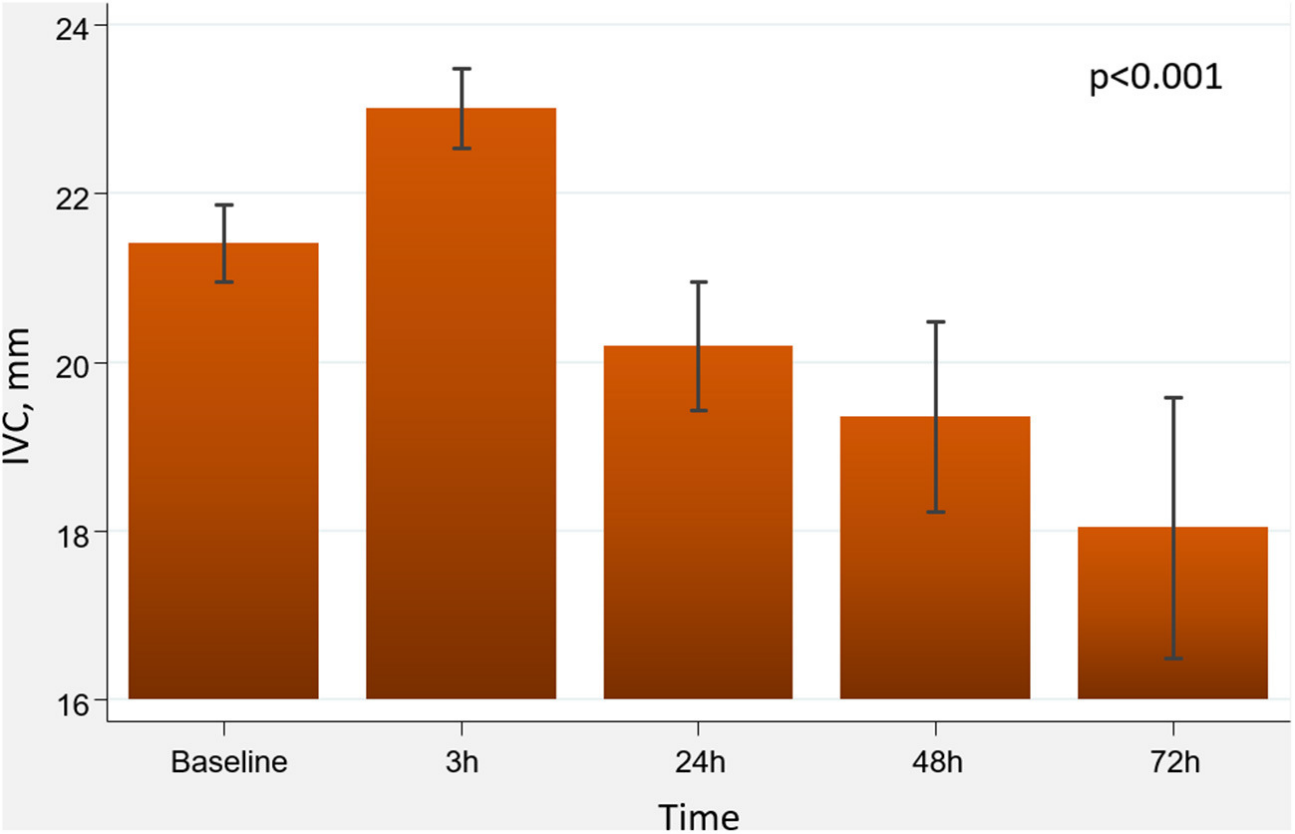
Changes in IVC diameter following administration of subcutaneous furosemide and venous leg compression. IVC, inferior vena cava.

**Figure 2:**
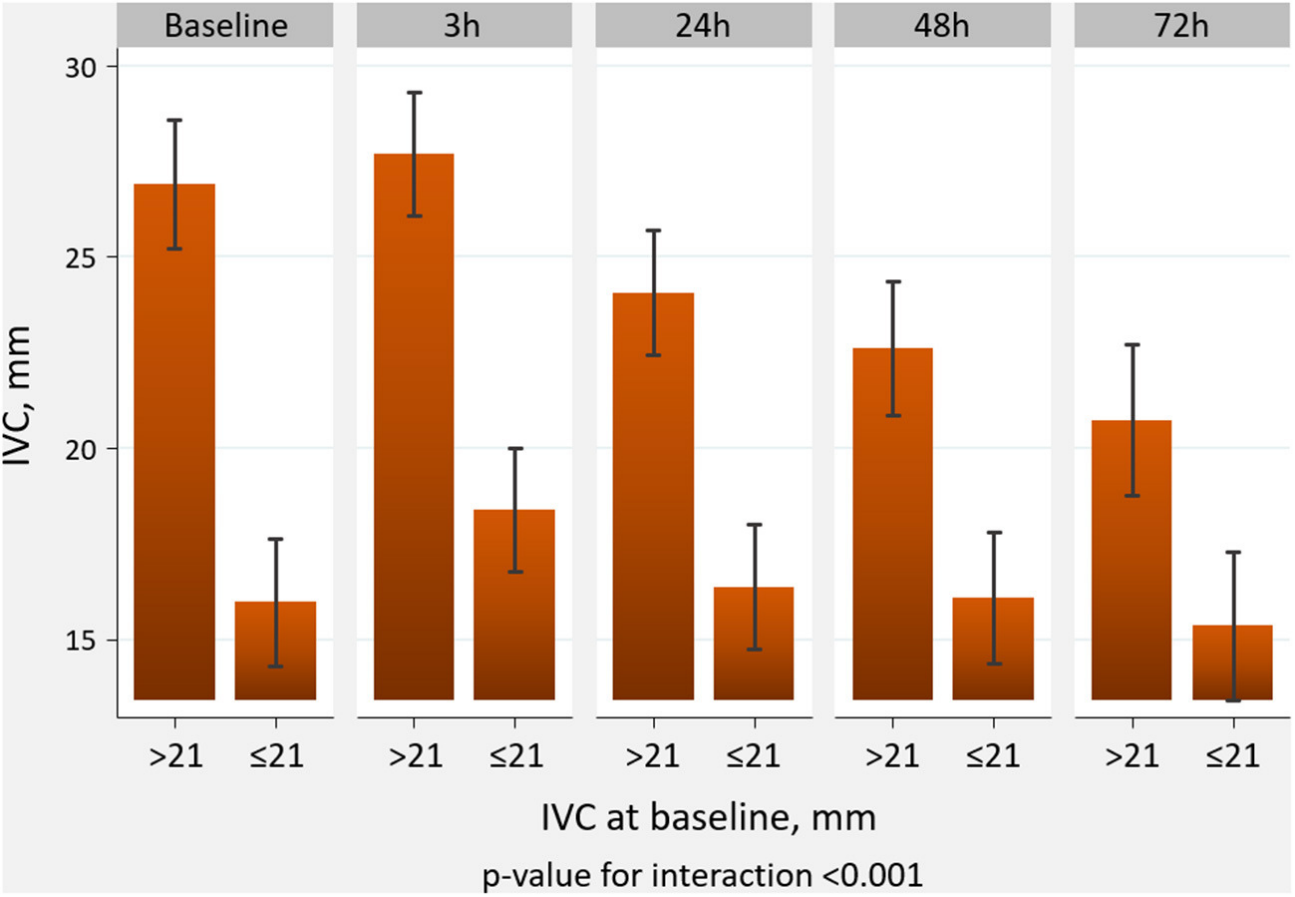
Effect of venous compression on the trajectory of IVC diameter across IVC status at baseline. IVC, inferior vena cava.

Following administration of subcutaneous furosemide and VLC, we found a significant increase in 3-h IVC diameter (ΔIVC = 1.6 mm, 95% CI: 0.7 to 2.5; *p* < 0.001). A total of 9 patients (45%) displayed a 3-h increase in IVC ≥2 mm. Afterward, a stepwise decrease of IVC diameter was noticed at 24, 48, and 72-h (Figure 1). However, the effect of VLC on the short-term trajectory of IVC diameter was heterogeneous across IVC status at baseline (*p*-value for interaction <0.001). In patients in which IVC was below or equal to the median (≤21 mm), we found a greater increase in 3-h IVC diameter (ΔIVC = 2.4 mm, 95%CI: 1.0 to 3.8; *p* < 0.001). In those with IVC >21 mm, IVC did not significantly increase at 3-h (ΔIVC = 0.8 mm, 95% CI:−0.6–2.2; *p* = 0.611), as is shown in Figure 2. The number of patients that increased IVC at least 2 mm at 3-h was higher in those with baseline IVC ≤21 mm [7 (70%) vs. 2 (20%), *p* = 0.025]. At later time-points (24, 48, and 72-h), and compared to baseline values, we found a greater decrease in IVC diameters in those with dilated IVC (>21 mm), but a neutral effect in those with IVC ≤21 mm (Figure 2).

### Decongestion Following Leg Compression

In the whole sample, and compared to baseline values, we found a significant increase in natriuresis, weight reduction, and decreased peripheral edema during the first 72 h (Supplementary Figure 1). Likewise, dyspnea VAS significantly decreased at 72-h (ΔVAS = −3.4, 95% CI: −4.1–−2.6; *p* < 0.001). We did not find significant changes in 72-h NT-proBNP (ΔLnNT-proBNP = −0.05, 95% CI: −0.23–0.34; *p* = 0.718). Renal function decreased at 72-h (ΔGFR = −4.2, 95% CI: −7.7–−0.7; *p* = 0.019). We also found a significant SBP and heart rate reduction at 24-h with posterior recovery (Supplementary Figure 1).

### Short-term Increase in Intravascular Volume and Decongestion/Safety: The Role of Baseline IVC Diameter

**Figure 3:**
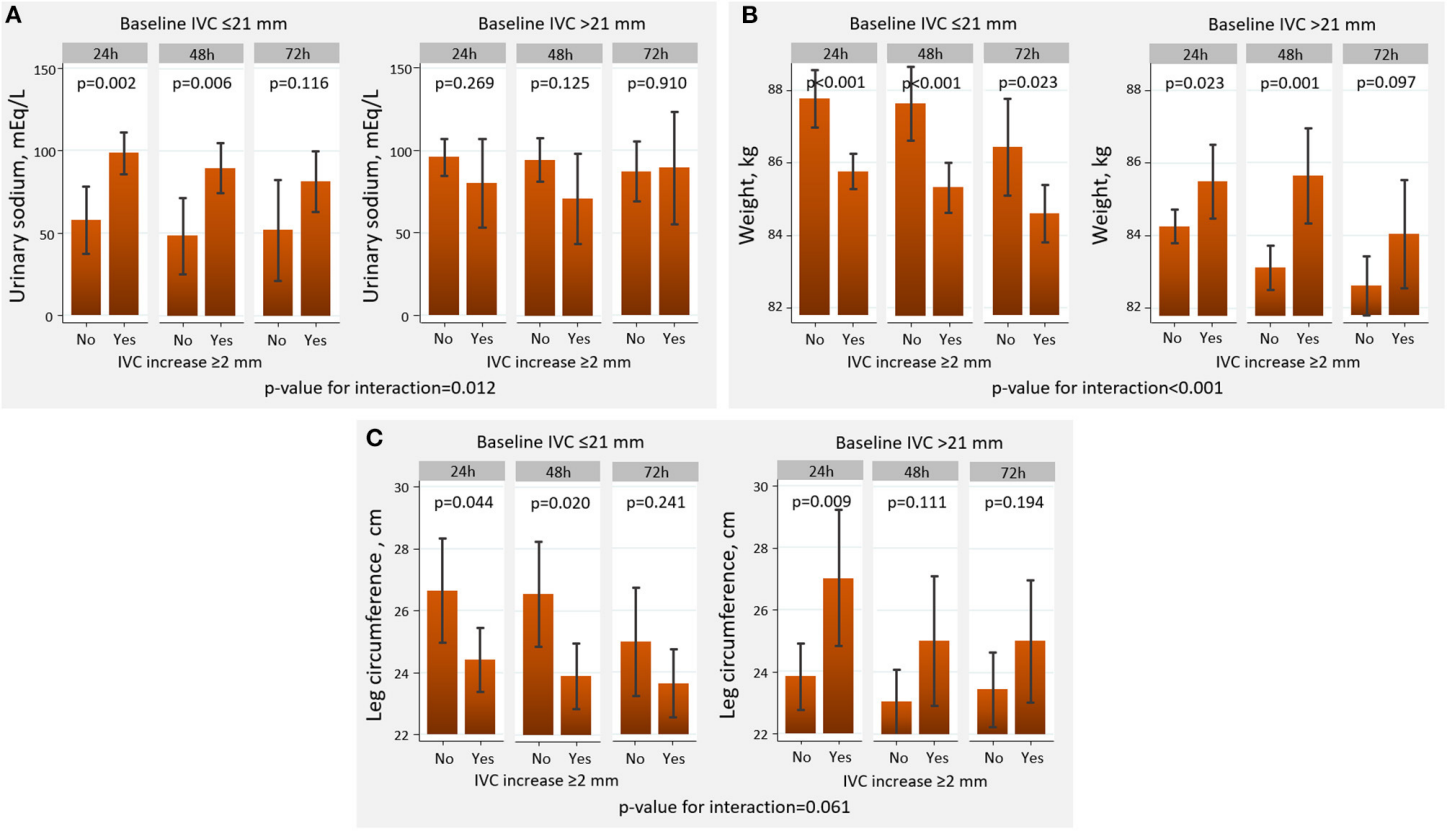
Changes in decongestion parameters across baseline IVC diameter and the presence of IVC increase at 3-h. **(A)** Urinary sodium. **(B)** Weight. **(C)** Leg circumference. IVC, inferior vena cava.

**Figure 4:**
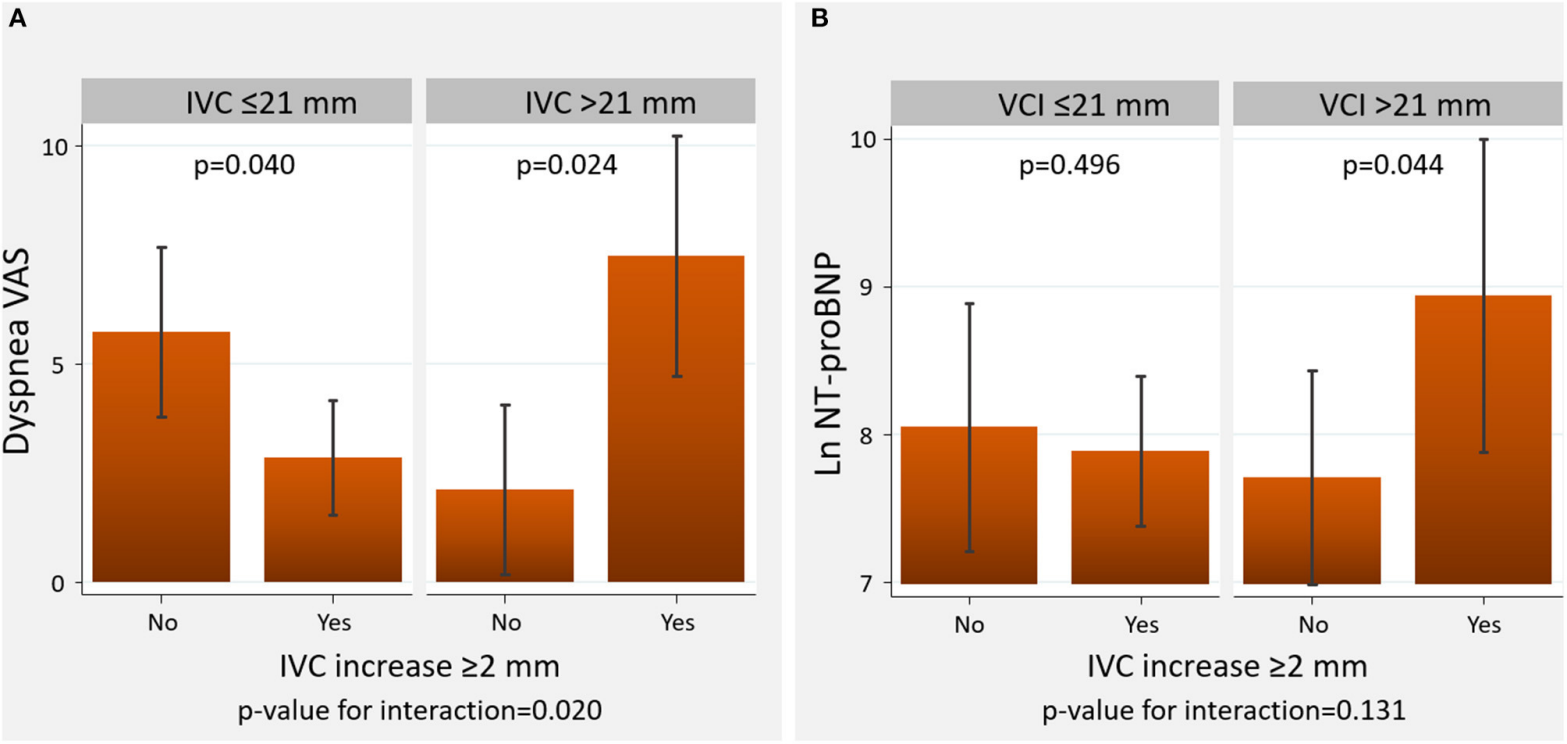
Changes in VAS and the logarithm of NT-proBNP across baseline IVC diameter and the presence of IVC increase at 3-h. **(A)** Visual analog scale. **(B)** Amino-terminal pro-brain natriuretic peptide. NT-proBNP, amino-terminal pro-brain natriuretic peptide, IVC, inferior vena cava; VAS, visual analog scale.

Overall, a short-term increase in IVC ≥2 mm was differentially associated with diuretic efficacy across baseline IVC diameter. An increase in IVC ≥2 mm was associated with a greater decongestion in those with baseline IVC ≤21 mm compared to those with dilated IVC (Figures 3, 4). All patients tolerated 72-h venous leg compression.

#### Urinary Sodium

A 3-hour increase in IVC ≥2 mm was associated with a greater natriuresis in those with IVC ≤21 mm but not in those with IVC >21 mm, in which most of the comparisons were neutral (p-value for interaction=0.012) (Figure 3A).

#### Weight

An increase in IVC ≥2 mm at 3-h was associated with a greater weight reduction when IVC ≤21 mm compared to those with IVC >21 mm (p-for interaction <0.001). In this latter group, intravascular refill was associated with higher weight (Figure 3B).

#### Peripheral Edema

The increase in IVC was borderline differentially associated with the resolution of edemas (*p*-value for interaction = 0.061). Intravascular refill led to a significant decrease in leg diameter in those with IVC ≤21 mm. Conversely, higher leg diameters were found in those with an increase in IVC ≥2 mm and baseline IVC >21 mm (Figure 3C).

#### Dyspnea VAS

IVC increase ≥2 mm at 3-h was associated with resolution of dyspnea in those with IVC ≤21 mm at 72-h, but not in those with IVC >21 mm (*p*-value for interaction=0.020). In this latter group, IVC increase ≥2 mm was associated with greater dyspnea at 72 h (Figure 4A).

#### NT-ProBNP

The relationship between an increase in IVC and 72-h NT-proBNP did not significantly differ across IVC status at baseline (*p*-value for interaction=0.131). However, we found higher LnNT-proBNP values in those with increased 3-h IVC and plethoric IVC at baseline (Figure 4B).

#### Estimated Glomerular Filtration Rate

**Figure 5:**
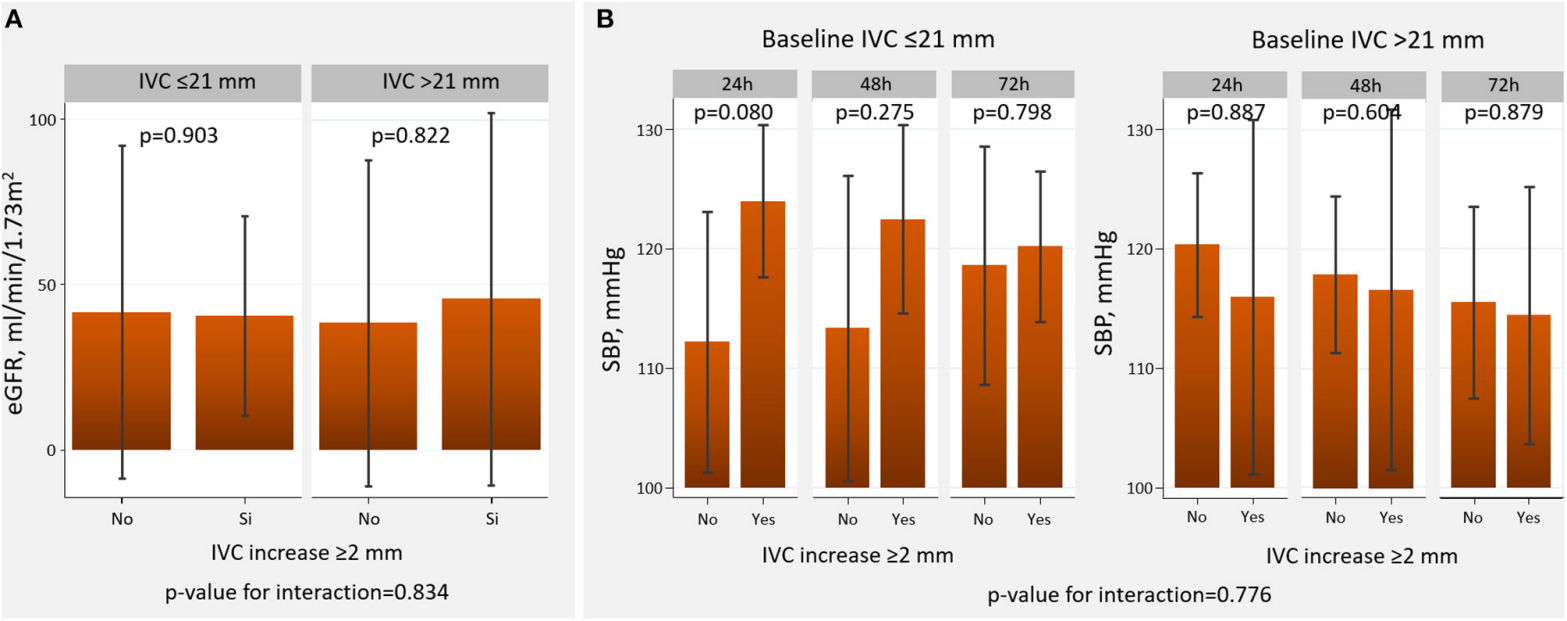
Changes in eGFR and SBP across baseline IVC diameter and the presence of IVC increase at 3-h. **(A)** Estimated glomerular filtration rate. **(B)** Systolic blood pressure. eGFR, estimated glomerular filtration rate; IVC, inferior vena cava; SBP, systolic blood pressure.

IVC 3-hour increase ≥2 mm was not differentially related to 72-h eGFR across baseline IVC (*p*-value for interaction = 0.835), as is presented in Figure 5A.

#### Systolic Blood Pressure

Short-term IVC increase ≥2 mm was not associated with a differential effect across baseline IVC (*p*-value for interaction = 0.776) (Figure 5B).

Summary of the main findings is presented in a central illustration.

## Discussion

**Figure 6:**
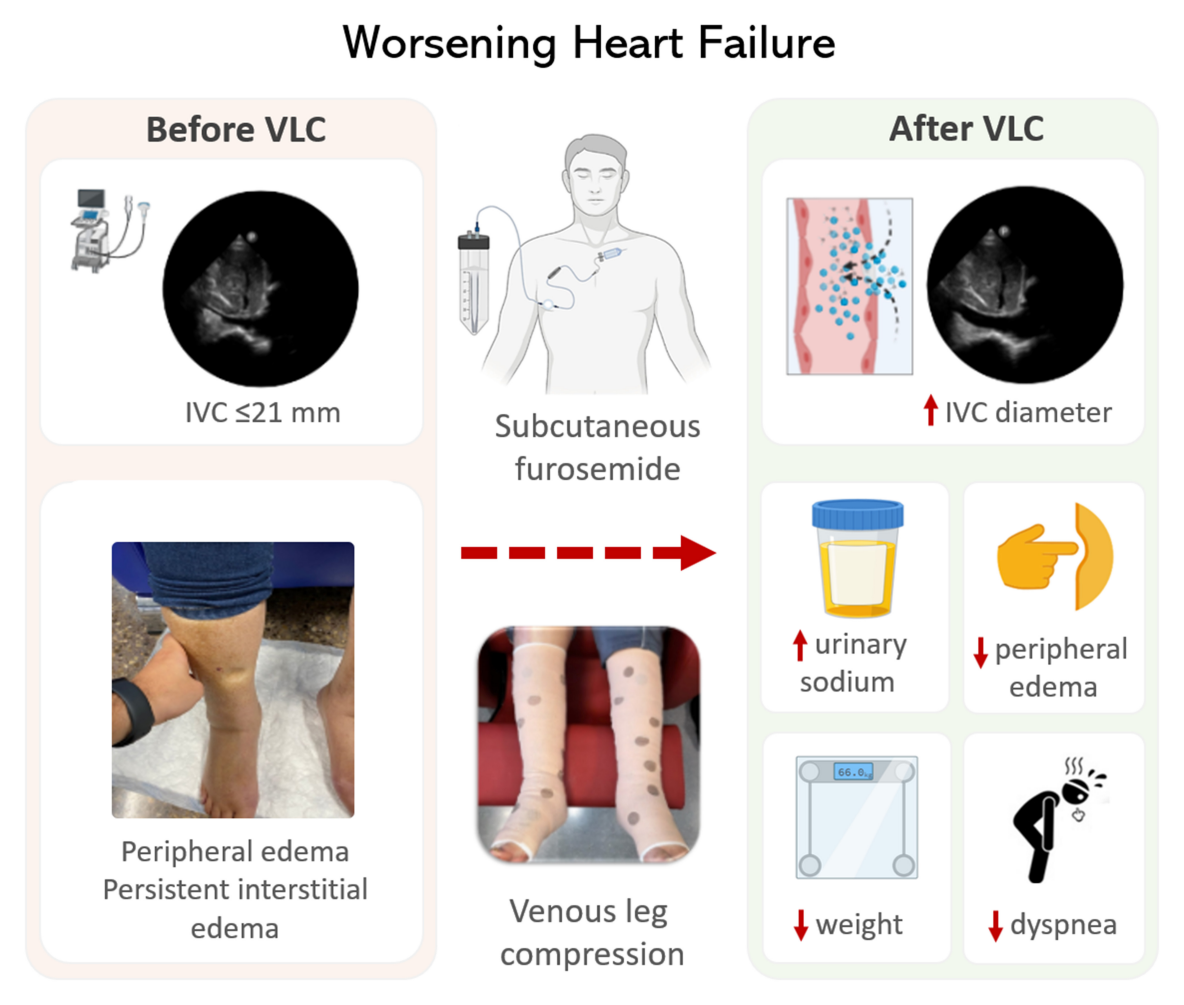
Central illustration. The use of venous leg compression in congestive heart failure. IVC, inferior vena cava.

We found that short-term VLC using elastic bandages may be useful for enhancing diuretic response in patients with congestive WHF treated with subcutaneous furosemide and absence of intravascular congestion (IVC ≤21 mm) at baseline. Conversely, it seems to have no role in those with intravascular congestion (Figure 6).

### Diuretic Response: The Role of Extravascular Congestion and Intravascular Refill

Parenteral loop diuretic agents are the mainstay of the treatment of patients with congestive WHF (3, 4, 8). Despite their ubiquitous use in daily clinical practice, the evidence base for the appropriate use of these agents remains largely empirical (3, 4, 8). Clinical trials are scarce, and most of them have resulted in non-conclusive results (3, 4, 8, 12, 13). Although congestion explains most symptoms and signs in decompensated patients, the severity and body distribution are largely heterogeneous (1, 2, 14, 15). Clinical proxies of congestion have also shown a limited ability for profiling the congestive phenotype in WHF (16), a fact that may easily explain the disappointing findings in several randomized clinical trials. Thus, there is consensus about the need to improve the assessment of HF congestion by using a multiparametric approach, including imaging, biomarkers, and clinical parameters (15, 16).

Most of the volume overload, especially at more advanced phases of the disease, corresponded to tissue and not circulatory congestion (17). Patients with predominant tissue congestion identified a subset of patients with lower diuretic efficiency and at higher risk of diuretic resistance (3, 4). Traditional depletive strategies mainly play a role in controlling intravascular congestion (3, 4), while effective treatment strategies for targeting tissue/extravascular congestion remain a clinical challenge (3, 4). To manage tissue congestion, different approaches aiming to facilitate the intravascular volume recruitment (transferring water from extravascular to an intravascular compartment) by increasing plasmatic osmolarity are used without robust evidence. These strategies included the infusion of loop diuretic together with a saline hypertonic solution or albumin (5, 6, 18, 19). Another approach consists of using pharmacological agents such as SGLT2i or tolvaptan that increase urine-free water elimination (4, 20).

This study postulates that VLC in patients with WHF, evident tissue congestion, and absence of intravascular congestion, might be a widely available therapeutic alternative for short-term intravascular compartment expansion. Interestingly, we found that intravascular expansion following VLC was greater in those with normal-low intravascular volume, and, in this subset of patients, it was positively associated with a greater decongestion. On the contrary, this maneuver appears futile or even not recommended in cases with circulatory volume overload.

### Previous Studies

To date, studies of VLC mainly focused on the treatment of chronic lower limb edema and ulcer, and few studies have been performed in congestive HF (7). Moreover, VLC in patients with severe and WHF appears as a contraindication in the guidelines dedicated to leg ulcer treatment (21), as a sudden movement of a large amount of blood from lower limbs veins could lead to a rapid increase in preload and afterload, precipitating pulmonary edema (7, 22). However, a contemporary report has suggested this strategy as safe in patients with venous ulcers and HF (23).

Among available studies in patients with HF, different populations have been analyzed. Dereppe et al. performed an invasive measurement of venous pressures using a Swan-Ganz catheter in 11 patients with HF (5 with chronic HF and 6 with acute myocardial infarction). After VLC, a significant increase in right auricular, pulmonary artery, and pulmonary wedge pressures was observed, and the values returned to baseline 30 min after finishing compression (24). Brain et al. also reported, in 15 patients with moderate to severe HF, that pneumatic VLC was associated with an increase in both mean right atrial pressure and pulmonary pressure, which did not translate into significant changes in left-sided heart function (25). However, Wilputte et al. observed, in 5 patients with HF and NYHA class III and IV that simultaneous bandage VLC and muscle contraction induced a significant increase in the right arterial pressure and a transient deterioration of the right and left ventricular function (26).

More recently, the effects of VLC with elastic bandages compared to hypertonic albumin on diuretic efficiency were evaluated in a large retrospective cohort of patients (*N* = 1147) with volume overload and diuretic resistance during the de-escalation phase of sepsis resuscitation (27). The use of elastic bandages was associated with superior diuretic efficiency than hypertonic albumin solution, despite lower baseline serum albumin levels in those receiving elastic bandages (27). Unfortunately, none of the prior works evaluated the effect of VLC across the intravascular status.

### Clinical Feasibility

In case of safety and efficacy confirmation, VLC by using elastic bandages is a widely available, simple, well-tolerated, and cheap intervention that may be easily translated into daily clinical practice.

### Further Studies

These findings underscore (a) the heterogeneous pathophysiology of congestion in WHF, (b) the complexity of managing congestion in HF, and (c) the need for moving toward a more precise medicine when tackling congestion in HF. Larger and more controlled studies are required to confirm current findings and unravel whether VLC may have a clinical role in managing patients with congestive HF with predominant tissue but not intravascular congestion.

### Limitations

First, it is a one-arm small pilot study with the absence of a control group. Further controlled studies are required comparing the effect of VLC plus parenteral administration of furosemide vs. parenteral administration of furosemide only. Second, the patients evaluated were older, with predominant preserved ejection fraction and features of advanced HF. Thus, current findings cannot be extrapolated to milder forms of the disease and those with predominantly left ventricular systolic dysfunction. Third, this study has the inherent limitations of the small number of participants. As such, we cannot discard that the neutral finding on some exposures may be due to low statistical power (Type II error). Fourth, the patients here included were those with ambulatory WHF. Leg compression's feasibility, efficacy, and safety should also be tested in hospitalized patients. Finally, the diuretic strategy used here was the subcutaneous administration of furosemide. Further studies should confirm current findings using intravenous administration of loop diuretics and better define the causal contribution of VLC in intravascular refilling.

## Conclusions

VLC treatment is safe in patients with congestive WHF. Treatment with VLC and subcutaneous furosemide was associated with a greater 72-h decongestion when IVC at baseline is within normal values. Conversely, it appears not to have a role in increasing diuretic response in those with WHF and dilated IVC at presentation.

## Data Availability Statement

The raw data supporting the conclusions of this article will be made available by the authors, without undue reservation.

## Ethics Statement

The studies involving human participants were reviewed and approved by Comité de Investigación del Instituto de investigación del Hospital Clínico Universitario de Valencia. Written informed consent to participate in this study was provided by the participants' legal guardian/next of kin.

## Author Contributions

JC and GM: conceptualization, data curation, investigation, methodology, project administration, validation, visualization, writing–original draft, and writing–review and editing. RE, ES, and AB-G: data curation, investigation, methodology, validation, visualization, and writing–review and editing. CS, AMo, AC, and AMa: data curation, visualization, and writing–review and editing. EN: formal analysis, investigation, methodology, resources, software, supervision, validation, and writing–review and editing. JN: conceptualization, formal analysis, funding acquisition, investigation, methodology, project administration, resources, software, supervision, validation, visualization, writing–original draft, and writing–review and editing. All authors contributed to the article and approved the submitted version.

## Funding

This work was supported by grants from the Ministry of Economy and Competitiveness, Instituto Carlos III (PI20/00392), CIBER Cardiovascular (16/11/00420 and 16/11/00403). The authors have no other funding, financial relationships, or conflicts of interest to disclose relative to this work.

## Conflict of Interest

The authors declare that the research was conducted in the absence of any commercial or financial relationships that could be construed as a potential conflict of interest.

## Publisher's Note

All claims expressed in this article are solely those of the authors and do not necessarily represent those of their affiliated organizations, or those of the publisher, the editors and the reviewers. Any product that may be evaluated in this article, or claim that may be made by its manufacturer, is not guaranteed or endorsed by the publisher.
