# Supplementary figures and images for: Venous Leg Compression for Tissue Decongestion in Patients With Worsening Congestive Heart Failure
Source: Front Cardiovasc Med. 2022 Jul 8;9:847450. doi: 10.3389/fcvm.2022.847450 (PMC9304621; doi:10.3389/fcvm.2022.847450)

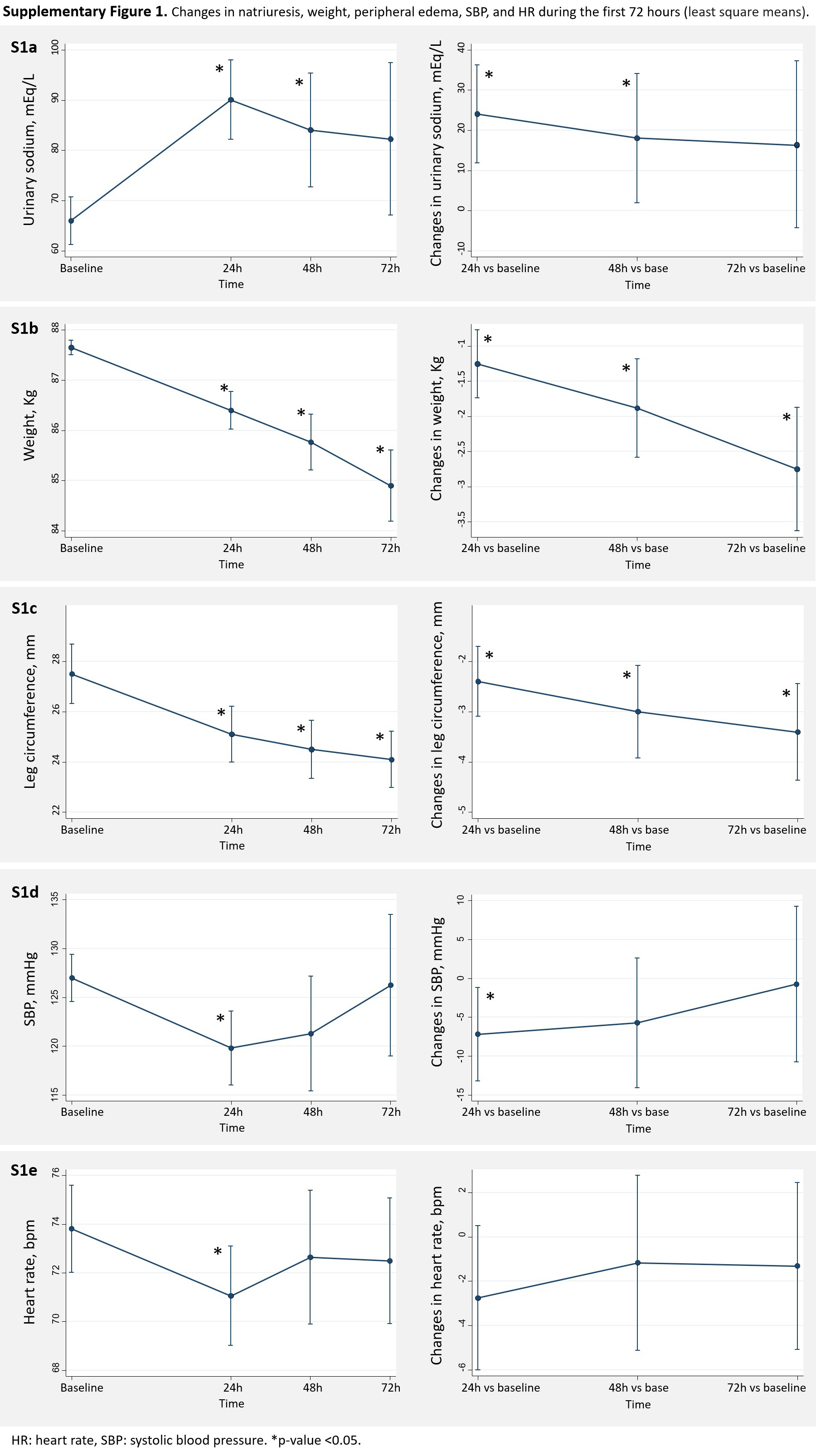

Supplement: Supplementary file 1 [file Image_1.JPEG]
